# Detecting Compensatory Growth in Silviculture Trials: Empirical Evidence From Three Case Studies Across Canada

**DOI:** 10.3389/fpls.2022.907598

**Published:** 2022-05-06

**Authors:** Chao Li, Hugh Barclay, Shongming Huang, Bernard Roitberg, Robert Lalonde, Nelson Thiffault

**Affiliations:** ^1^Canadian Wood Fibre Centre, Canadian Forest Service, Edmonton, AB, Canada; ^2^Pacific Forestry Centre, Canadian Forest Service, Victoria, BC, Canada; ^3^Alberta Agriculture, Forestry and Rural Economic Development, Edmonton, AB, Canada; ^4^Department of Biological Sciences, Simon Fraser University, Burnaby, BC, Canada; ^5^Department of Biology, University of British Columbia, Okanagan, BC, Canada; ^6^Canadian Wood Fibre Centre, Canadian Forest Service, Québec City, QC, Canada

**Keywords:** density management, overcompensation, plantation spacing, thinning operation, wood fiber value simulation model

## Abstract

Compensatory growth (CG) appears common in biology and is defined as accelerated growth after experiencing a period of unfavorable conditions. It usually leads to an increase in biomass that may eventually equal or even surpass that of sites not experiencing disturbance. In forestry, with sufficient time the stand volume lost in a disturbance such as a thinning operation could match or even exceed those from undisturbed sites, respectively called exact and overcompensation. The forest sector could benefit from enhanced productivity and associated ecosystem services such as carbon storage through overcompensation. Therefore, detection of CG in different types of forests becomes important for taking advantage of it in forest management. However, compensatory growth has not been reported widely in forestry, partially due to the paucity of long-term observations and lack of proper indicators. Legacy forest projects can provide a suitable data source, though they may be originally designed for other purposes. Three case studies representing different data structures of silviculture trials are investigated to evaluate if compensatory growth is common in forest stands. Our results showed that compensatory growth occurred in all three cases, and thus suggested that the compensatory growth might indeed be common in forest stands. We found that the relative growth (RG) can serve as a universal indicator to examine stand-level compensatory growth in historical long-term silviculture datasets. When individual tree-based measurements are available, both volume and value-based indicators can be used in detecting compensatory growth, and lumber value-based indicators could be more sensitive in detecting overcompensation.

## Introduction

Forest productivity representing long-term potential in forest dynamics is one of the major concerns and indicators in achieving sustainable forest management (Crow et al., 2006). It is also positively correlated with many ecosystem services such as carbon sequestration and storage (Kurz et al., 1992, 2009; Pöyry, 2018). Enhancement of forest productivity will thus not only produce more wood for necessities of human society, but also present a natural remedy against climate change through altering regional carbon budgets. Exploring the conditions under which enhanced forest productivity could occur is important in this regard.

Considerable efforts have been devoted to enhancing forest productivity through silviculture (Natural Resources Canada, 1995; D’Amato et al., 2017), among which is stand density management that aims at determining the optimal stand density leading to maximizing management goals (Drew and Flewelling, 1979; Newton, 1997). Numerous experiments have been conducted to determine the effectiveness of density management for identifying best practices. Diverse results have also been reported in the literature: some are consistent, but others appear inconsistent. For example, there is a consensus that precommercial thinning (PCT) can result in faster growth of diameter and height in trees than that from untreated sites (e.g., Smith, 1986; Weetman and Mitchell, 2013). Bose et al. (2018) showed that in four major softwood species in North America [i.e., loblolly pine (*Pinus taeda* L.), coastal Douglas-fir (*Pseudotsuga menziesii* Mirbel), red spruce (*Picea rubens* Sarg.), and balsam fir (*Abies balsamea* L.)], tree volume growth was 31% higher in thinned stands relative to unthinned stands on average, irrespective of species and tree size. However, in long-term observations at the stand level, consequences of PCT are inconsistent: some appeared to have lower stand productivity compared to controls (e.g., Harrington and Reukema, 1983), but the reverse has also being reported (e.g., Warrack, 1979; Pitt and Lanteigne, 2008; Schütz et al., 2015). These inconsistent results unavoidably create difficulties in management decisions. Here, the great puzzle is how such inconsistent and diverse results occur.

The framework of compensatory growth (CG) might help explaining these inconsistent results. For example, Li et al. (2018) evaluated the different growth responses, including overcompensation, from the Shawnigan Lake PCT and fertilization trial in British Columbia (Canada), in which stand gross volumes from most treated sites exceeded those from controls 40 years after initial treatments. A descriptive model was then developed to predict the number of years required to reach an exact compensation, or the compensatory-induced-equity (CIE), under different combinations of PCT and fertilization. This unique dataset offers the opportunity to generalize the results to other species, site conditions, and geographical regions (Li et al., 2020).

Compensatory growth was defined as “the ability of an organism to grow at an accelerated rate following a period of food shortage or a decline in reproductive weight” (Mangel and Munch, 2005), and is widely observed in plants and animals as reviewed by Li et al. (2021). Despite the concept of CG being relatively new in tree biology and forestry, it has been commonly reported for other organisms; the earliest observations can be traced back about a century ago in rats and cotton crops (Osborne and Mendel, 1915, 1916; Eaton, 1931). It has also been observed in grasslands (McNaughton, 1985), fishes (Ali et al., 2003), fast-growing willow (Guillet and Bergström, 2006), balsam fir (Pitt and Lanteigne, 2008), aspen (*Populus tremuloides* Michx.) seedlings (Erbilgin et al., 2014), and Douglas-fir (Schütz et al., 2015; Li et al., 2018). In forestry, CG of trees is a special case of growth response that characterizes the process of released growth of crop trees after thinning operations, or other partial disturbances that maintain a forest cover. It reflects the accelerated growth induced by the redistribution of space, light, nutrients, and water to the trees surviving from the disturbance (e.g., thinning). Nevertheless, the CG process has not been shown to display a single fixed pattern; it appears to depend on species, social environment, seasonal development, temperature, environmental resource availability, and physiological factors such as internal state and age (Mangel and Munch, 2005).

Three main types of CG have been demonstrated along a continuum: (1) under compensation, in which stand volumes of treated sites are lower than those from control sites; (2) exact compensation, or CIE, in which stand volumes of treated sites are the same as those from control sites; and (3) overcompensation, in which stand volumes of treated sites are higher than those from control sites (Belsky, 1986; Maschinski and Whitham, 1989; Whitham et al., 1991; Li et al., 2020, 2021). In the short term, CG is expressed as an accelerated annual increment of diameter and height after thinning operations. When this trend persists for a longer term, overcompensation, the most desirable outcome from silviculture research, becomes possible, as observed for the costal Douglas-fir in the Shawnigan Lake trial (Li et al., 2018). Thus, when accelerated growth occurs over a certain period, under compensation may proceed to exact compensation, and later to overcompensation.

Compensatory growth can be seen as a component of the total Density-Dependent (D-D) response to disturbances such as fire, insect attack, death of senescent trees, and PCT. The D-D response indicates that population size may oscillate back and forth across the mean population size at carrying capacity for some time (Varley et al., 1973) when the response is over-compensatory. For trees, the status of volume can change from under-compensation to CIE, and later to over-compensation, so that the age and status of the tree’s growth cycle need to be specified. From an evolutionary biology perspective, CG can also be related to the concept of life-history theory (LHT), which “attempts to understand how natural selection designs organisms to achieve reproductive success, given knowledge of how selective factors in the environment (i.e., extrinsic mortality) and factors intrinsic to the organism (i.e., trade-offs, constraints) affect survival and reproduction” (Fabian and Flatt, 2012). LHT starts with the basic principle of evolutionary biology that natural selection favors the strategies of organisms that maximize reproductive success. One can then use LHT to find the optimal resource or energy allocation strategy among growth, reproduction, and maintenance of individual trees. Nevertheless, the concept of CG appears straightforward and easy to understand without requiring specific knowledge of a given speciality.

Li et al. (2021) summarized possible gains that different industries can benefit from with CG research: (1) increased productivity directly from overcompensation; and (2) indirectly from reduced costs associated with production and related operations. To determine the best strategy for taking advantage of CG research, forest managers and researcher need to understand the CG patterns and status of managed forests. For example, if overcompensation can be expected, the direct benefit should be targeted first, due to enhanced productivity that will benefit both increased wood supply and improved ecosystem services, without excluding the benefits from the indirect aspects. From an interdisciplinary point of view, there is a possibility of higher productivity potential from observed productivity of natural stands, if manipulated properly (Li et al., 2021). Therefore, detecting CG patterns and status in forests, especially determining whether overcompensation is reachable, is important for the forest sector in designing future research focus.

Despite the fact that CG has been reported as a common phenomenon in both plants and animals, and that different industries have benefitted greatly from CG research, direct evidence of CG in tree biology and forestry has not been as widely reported as in other fields, and the beneficial applications of CG research results are still limited. It may be unrealistic or infeasible to rely on efforts of designing and implementing new experiments for answering related questions, due to the long lifespan of trees, especially in sub-boreal and boreal ecosystems (several decades). However, revisiting datasets from historical long-term legacy projects might provide useful clues, though these experiments were not originally designed for this purpose. Owning to the different purposes of these trials, different data structures could provide different measured variables at different measurement intervals. Therefore, finding the common variables, either measured or derived, as indicators of CG is a major task in detecting CG in different tree species and different regions.

We hypothesized that CG is common at tree and stand levels. If this hypothesis were true, one could expect that plenty of empirical evidence should exist in historical forestry datasets. To examine this hypothesis, proper methodology needs to be developed to detect CG from historical datasets. The research questions raised here include “what indicators could be used for identifying CG?”, and “which one can serve as a universal indicator of CG?” In this study, we use three published datasets as case studies to identify and demonstrate potential indicators of CG, in addition to stand gross volume, and to identify a universal indicator that could be used for detecting CG from a wide range of historical forestry datasets.

Historical silviculture datasets usually include stand variables, which are calculated from tree data, the standard format for silviculture research. We will show that relative growth (RG) can serve as a universal CG indicator for both tree and stand level variables. When tree level variables are available, many can serve as indicators of CG, although value-based indicators might be more sensitive to overcompensation than volume-based indicators.

## Materials and Methods

### Study Areas and Data Sources

**Figure 1:**
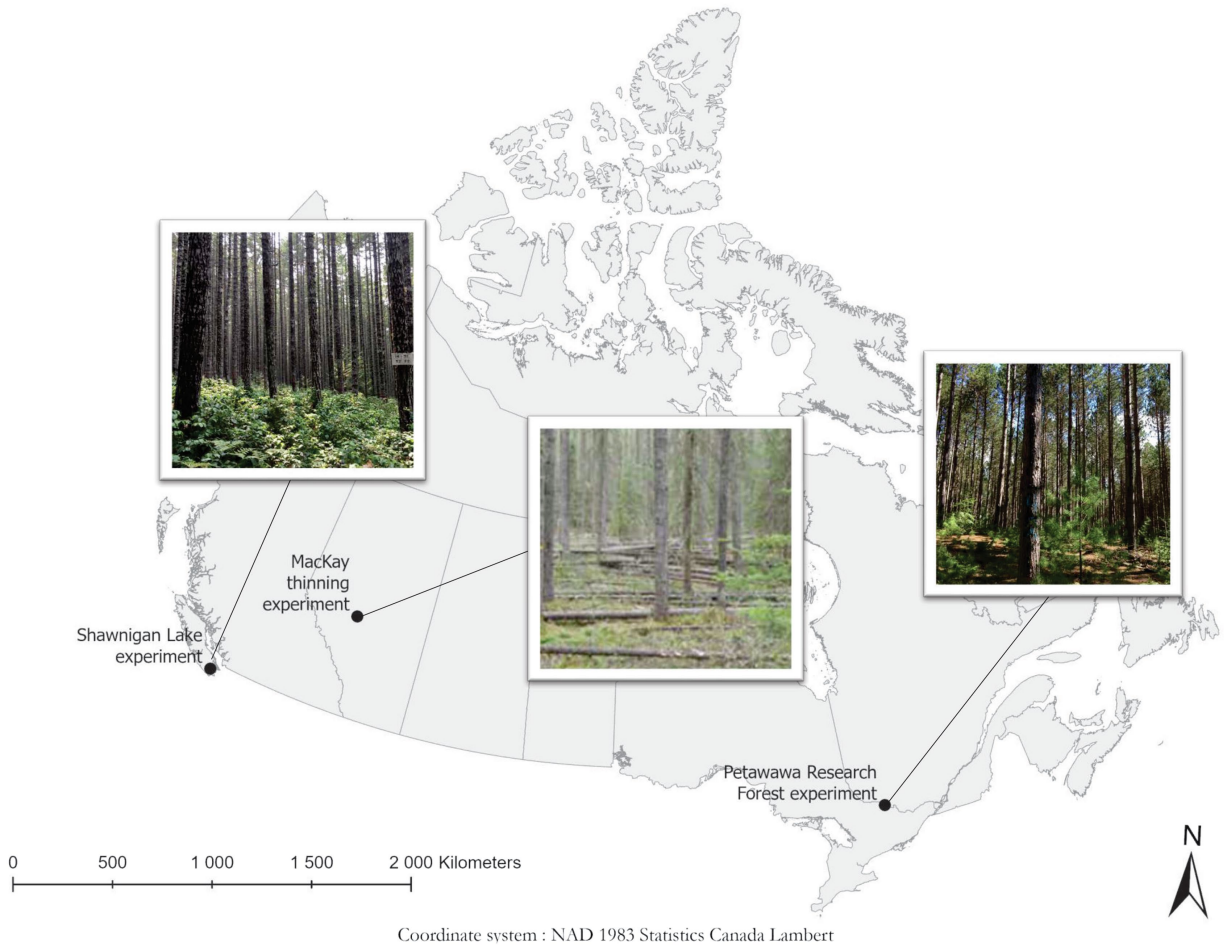
Locations of the three case studies in Canada, with a photograph of a thinned plot from each site. Photos of Shawnigan and Petawawa are from N. Thiffault. The photo of MacKay is from B. Joss and J. Salvail, as used in Stewart and Salvail (2017).

Figure 1 illustrates the locations of our three case studies. Key information about the case studies is summarized in Table 1.

**Table 1:** Summary of the three case studies in Canada.

Case study	Location	Size of study site	Species	Year of establishment	Site index at age 50	Experiment design	Reference
MacKay thinning experiment	Foothill Alberta (lat. 53°32.7″N; long. 115°32.3″W)	1.5 ha	Lodgepole pine (*Pinus contorta* Douglas)	1954	18 m	Thinning at 22-year old stands of fire origin; treatments of spacing density of 4,330, 2,990, 1,680, and 750 stems/ha and a control arranged in a randomized complete block design with multiple replicates; measured in 1954, 1960, 1969, 1979, 1989, 1996, 2003, 2008, and 2013 with a measurement plot size of 0.08 ha each in block A, B, and C, and of 0.30 ha in block D; measured for DBH; 1.3 m for all living trees.	Stewart et al., 2006; Stewart and Salvail, 2017
Petawawa research forest experiment	Petawawa Research Forest in Chalk River, Ontario, Canada (lat. 46°00′0″N; long. 77°26′0″W)	10 stands of about 1.6 ha each	Red pine (*Pinus resinosa* Aiton)	1953	24 m	Established on abandoned farmland; a factorial design of six initial spacings of 1.2, 1.5, 1.8, 2.1, 2.4, and 3.0 m were randomly allocated to 10 stands; In 1962, two 0.101 ha sampling plots were established for each spacing and measured periodically; repeated commercial thinning in 1992, 2002, and 2013, bringing BA down to the same original residual BA target value of 37.9 m^2^/ha; DBH and TH before each of the thinning entries were measured for the permanent sampling plots (0.08–0.101 ha) established in each spacing-thinning treatment combination; DBH was measured for every tree in each sample plot, but TH was only measured for a subsample of trees (19–76% of live stems) through a range of diameters.	Thiffault et al., 2021
Shawnigan lake experiment	Shawnigan Lake, BC (lat. 48°38′13″N; long. 123°43′16″W)	50 ha	Coastal Douglas-fir (*Pseudotsuga menziesii* Mirbel)	1971–1972	21 m	Thinning and fertilization treatments on 23-year old plantation with averaged 7.6 cm DBH and 8.6 m TH; a randomized 3 × 3 factorial design comprising three levels of thinning (T0, T1, and T2 corresponding to 0, 1/3, and 2/3 BA removal, respectively) and three levels of fertilization (F0, F1, and F2 corresponding to 0, 224, and 448 kg N/ha of fertilizer, respectively); multiple replications measured in plots with a size of 0.0405 ha each, surrounded by a 15-m-wide treated buffer; measured in 1, 3, 6, 9, 12, 15, 24, 32, and 40 years after initial treatments.	Crown and Brett, 1975; Omule et al., 2011; Li et al., 2018

*DBH, diameter at breast height (1.3 m); TH, tree height; and BA, basal area*.

#### The MacKay Thinning Experiment

Lodgepole pine (*Pinus contorta* Douglas) is a major economic species on the eastern slopes of the Canadian Rocky Mountains; it is commonly grown over rotations of 70–150 years (Stewart et al., 2006). Since 1938, the Canadian Forest Service (CFS) has established dozens of silvicultural field studies involving this species, ranging from simple trials of a single thinning prescription, to complex levels of growing stock installations replicated on sites of differing productivity or aspect, to factorial experiments of thinning and fertilization. Although most of these studies have been discontinued, a number of them remain undisturbed and adequately documented to justify their continuation as active research sites for intensive management of the foothills lodgepole pine resource (see Figure 1 and Table 1 for location and summary info).

Stewart and Salvail (2017) evaluated the effects of different PCT treatments on the growth of lodgepole pine using four study sites, including the MacKay thinning experiment established almost 70 years ago. The MacKay thinning experiment dataset contains the complete records of untreated plots corresponding to different treatments that allows calculation of relative volume growth at each measurement.

The original objective of the experiment was to determine whether PCT of lodgepole pine could improve merchantable volume and quality at a young age and, in turn, shorten rotation and increase annual allowable cuts. Only stand density, mean diameter at breast height (DBH; 1.3 m), tree height (TH), and stand volume data were used in the current analysis.

#### The Petawawa Research Forest Experiment

Red pine (*Pinus resinosa* Aiton) is a widely planted species across North America exhibiting a faster growth rate compared with most native tree species in northern United States and southeastern Canada (Thiffault et al., 2021). Forest managers generally recognize that high planting densities can result in greater volume production at stand level, but that maximized individual tree size can be achieved at lower densities. Optimum planting densities and thinning intensities can be derived from density management diagrams, which are based on assumptions derived from the observations of unmanaged stands. However, empirical data from real stand density management treatments are expected to provide objective observations from managed stands (see Figure 1 and Table 1 for location and summary info).

Commercial thinning (CT) has recently attracted attention as one possible way of dealing with wood supply shortage, because it provides a means to utilize wood supply from immature stands and take advantage of low stumpage price. Compared with PCT, CT is an operation with notable immediate economic gain from harvested wood. CT can be applied multiple times for a stand to reach enhanced total stand productivity, which is calculated as the sum of final standing volume and cumulative volume harvested from multiple CT operations. Multiple CTs usually result in chainsaw shape for standing volume over time (Nyland, 1996). The chainsaw shape of stand productivity shows CG processes with multiple reductions caused by each of the CT operations. The key question is whether cumulative compensation overtakes or even exceeds the growth observed in control sites. Theoretically, thinning keeps the remaining trees vigorous and less prone to breakage, therefore when performed appropriately, the cumulative stand productivity from the CT stands could exceed that from control.

A recently published red pine dataset regarding a 30-year multiple CT trial under different spacing regimes (supplemental materials of Thiffault et al., 2021) was used for our second case study. The dataset contained mainly stand density, gross volume, and basal area at the stand level. The paired volume data structure allowed calculation of relative volume growth.

#### Shawnigan Lake Experiment

Coastal Douglas-fir is a long-lived species in northwest coast areas of North America; it can typically live more than 750 years in the absence of high-intensity fires or storms, and some have been known to live well over 1,000 years. Douglas-fir has also a long silviculture history in the region because of its high-value timber at minimum direct cost (Curtis et al., 1998). Plantation and silviculture of coastal Douglas-fir have been common in the Pacific Northwest (PNW) of United States and western Canada (see Figure 1 and Table 1 for location and summary info).

The original objective of the trial was to better understand tree growth processes and develop effective and environmentally acceptable thinning and fertilization practices for increasing wood yields (Crown and Brett, 1975). Only the measurements at 40-year after initial treatments were used in our current study.

### Evaluation Methods

#### Relative Growth

Relative growth was used as an indicator of the status of CG defined as the ratio of estimated variables from treated site, *V_treated_*, to untreated site, *V_untreated_* (Li et al., 2018). The ratio is presented as a percentage:


(1) $$RG=\left(V_{Treated}/V_{Untreated}\right)\times 100\%$$

A CIE is indicated when RG is 100%, i.e., the variable measured from the treated site equals to the one from the untreated site. Overcompensation is denoted when RG is greater than 100%, and undercompensation occurs when RG is less than 100%. Therefore, the status of CG can be seen as a point within a continuum as defined in Maschinski and Whitham (1989) and Whitham et al. (1991). In the current study, RG was used to measure the status of CG in volume- and value-based measurements.

Other alternative indicators could also be developed, such as the changes in mean periodic annual increment (mPAI), as suggested in Li et al. (2020). However, here we focus on the relative growth because of its simplicity and ease of calculation.

#### WFVSM Model

The Wood Fiber Value Simulation Model (WFVSM) was used to calculate both volumes and values of a forest inventory consisting of tree-based records (Li et al., 2017). A brief description of data flow and analytical procedure of WFVSM is as follows: the records are first categorized by species groups of softwood and hardwood, and followed by volume and value calculations for trees harvested for segregation to different types of treatment centers for different products, which include sawmill, pulp mill, veneer mill, plywood mill, biomass, bioenergy (heating values and electricity), carbon content, and some wood fiber attributes. These calculations were based on published relationships (e.g., Briggs, 1994), except for sawmill output, which was simulated using an industrial sawmill operation software, Optitek (Li et al., 2013; FPInnovations, 2014).

Optitek was originally designed for simulating every step of operations in a real sawmill when profiles of individual logs are scanned into the computer system as images. It also has an input option for an artificial log consisting of variables including log length and sweep, and diameters at both large and small ends. Taper equation of Kozak (1988) was used to calculate the diameters at both large and small ends for each log. The eight parameters of the Kozak equation for coastal Douglas-fir were obtained from the Forest Inventory Zones A, B, and C of British Columbia (BC Ministry of Forests, Lands and Natural Resource Operations, 2014).

A look-up-table was constructed for coastal Douglas-fir through multiple simulations using Optitek. Each row of the table represents a summarized sawmill output for a possible combination of DBH (at the interval of 1 cm) and TH (at the interval of 1 m). All the values including volumes and monetary values of lumber, chip, sawdust, and shavings, as well as planing and fixed costs are among the tallied simulation results.

The estimation of the values of each treated and untreated plot can then be guided by basic principle of forestry economics (Pearse, 1990):


(2) $$V_{Net}=V_{Revenue}-V_{Cost}$$

where *V_Net_* is the net value, *V_Revenue_* is the revenue, and *V_Cost_* is the associated cost. Both *V_Revenue_* and *V_Cost_* can be generated from different utilization of harvested wood, such as sawmill, pulp mills, veneer mills, plywood mills, and bio-refineries, as well as when harvested wood is used as a source of biomass, and carbon capture. In the sawmill operation, for instance, *V_Revenue_* can be expressed as


(3) $$V_{Revenue}=V_{Lumber}+V_{Chip}+V_{Sawdust}+V_{Shavings}+V_{Bark}$$

where *V_Lumber_*, *V_Chip_*, *V_Sawdust_*, *V_Shavings_*, and *V_Bank_* are values of lumber, chips, sawdust, shavings, and bark, respectively. *V_Cost_* can be estimated as


(4) $$V_{Cost}=V_{Drying}+V_{Planing}+V_{Fixed}$$

where *V_Drying_*, *V_Planing_*, and *V_Fixed_* are the costs of drying, planing, and fixed cost. Substituting Eqs. 3 and 4 into Eq. 2, we then have


(5) $$\begin{array}{l} V_{Net}=\left(V_{Lumber}+V_{Chip}+V_{Sawdust}+V_{Shavings}+V_{Bark}\right)\\ -\left(V_{Drying}+V_{Planing}+V_{Fixed}\right) \end{array}$$

### Statistical Analysis

The scattered RG data for different variables were smoothed as a surface to represent the trend of RG under different thinning (including PCT or CT) and fertilization treatments when applicable, using TableCurve 3D (SYSTAT Software Inc., 2002).

A two-way ANOVA (McDonald, 2014) was used to investigate whether the average stand commodity values could be significantly influenced by different levels of thinning and fertilization, as well as their interactions for the Shawnigan Lake dataset. ANOVAs were conducted using the car package in R v. 4.0.4 (Fox and Weisberg, 2019). We used *p* < 0.01 as a significance threshold.

## Results and Discussion

### Case Study 1 (Lodgepole Pine): Volume-Based Indicators Using Stand Level Data

Many documented long-term silviculture trials have been designed for studying density management, because density is perhaps the easiest variable to control in a forest stand. A common indicator used in reporting results from such trials is the gross volume at the stand level, which allows comparing the observations from treated vs. untreated sites. They represent an excellent data source for examining whether CG is expressed in forests. The relative volume growth represented in Equation 1 can be used to detect whether or not stands displayed CG, as demonstrated here.

The initial stand density in the study area varied between 9,763 and 11,888 stems/ha. These initial densities were much higher than for some other tree species, such as coastal Douglas-fir from the Shawnigan Lake trial (3,950 stems/ha, see the case study #3 below). In Blocks A, B, and C, six plots were established (thinned to a density of 4,330 stems/ha in plot 1; 2,990 stems/ha in plot 2, 4, and 5; and 1,680 stems/ha in plot 3). Block D had one plot thinned to a density of 750 stems/ha. As a result, the numbers of trees removed at the beginning for spacing design were very high (55–93%). In the control plots (plot 6 in Block A, B, and C), self-thinning was important (76–79% over 60 years). This suggested that thinning removed large numbers of trees that might otherwise have been lost in self-thinning, such as reported by Thiffault et al. (2021). Since plot 5 was thinned twice, the measurement was not included in the current study for the purpose of comparison.

Table 2 shows some statistics from the MacKay trial dataset. Among the 13 plots (except three control plots), eight of them reached relative volume growth of over 100% (exact or overcompensation), and five were below 100% (under compensation). Unless the stand density was too low to use the available resources efficiently, there is no reason to expect these plots will not catch-up with the control given sufficient time, since they already reached 85–97% of gross volumes of the control plots.

**Table 2:** Stand density, mortality, and maximal relative volume growth of the MacKay thinning trial in Alberta, Canada, in which naturally regenerated lodgepole pine (*Pinus contorta* Douglas) stands were submitted to a gradient of pre-commercial thinning intensity.

Treatment code^*^	Stand density (Stems/ha)		Mortality (%)			Max relative volume growth (%)
	1954	2013	Total	Thinning	Nature	
A1	4,330	1,593	85	60	25	104
A2	2,990	1,173	89	72	17	116
A3	1,680	840	92	84	8	97
A4	2,990	1,124	90	72	17	107
A6	10,762	2,462	77	–	77	100
B1	4,330	1,494	87	64	24	100
B2	2,990	1,082	91	75	16	86
B3	1,680	1,074	91	86	5	85
B4	2,990	1,396	88	75	13	98
B6	11,887	2,850	76	–	76	100
C1	4,330	1,457	85	56	29	109
C2	2,990	1,334	86	69	17	125
C3	1,680	1,037	89	83	7	102
C4	2,990	1,569	84	69	15	103
C6	9,762	2050	79	–	79	100
D1	750	602	94	92	2	95

*Maximum relative volume growth after a 60-year experimental period was calculated as the ratio of the volume obtained in the treated vs. control stands (see Eq. 1)*.

* *Treatment code represents the combination of block name and plot number at the MacKay thinning trial*.

**Figure 2:**
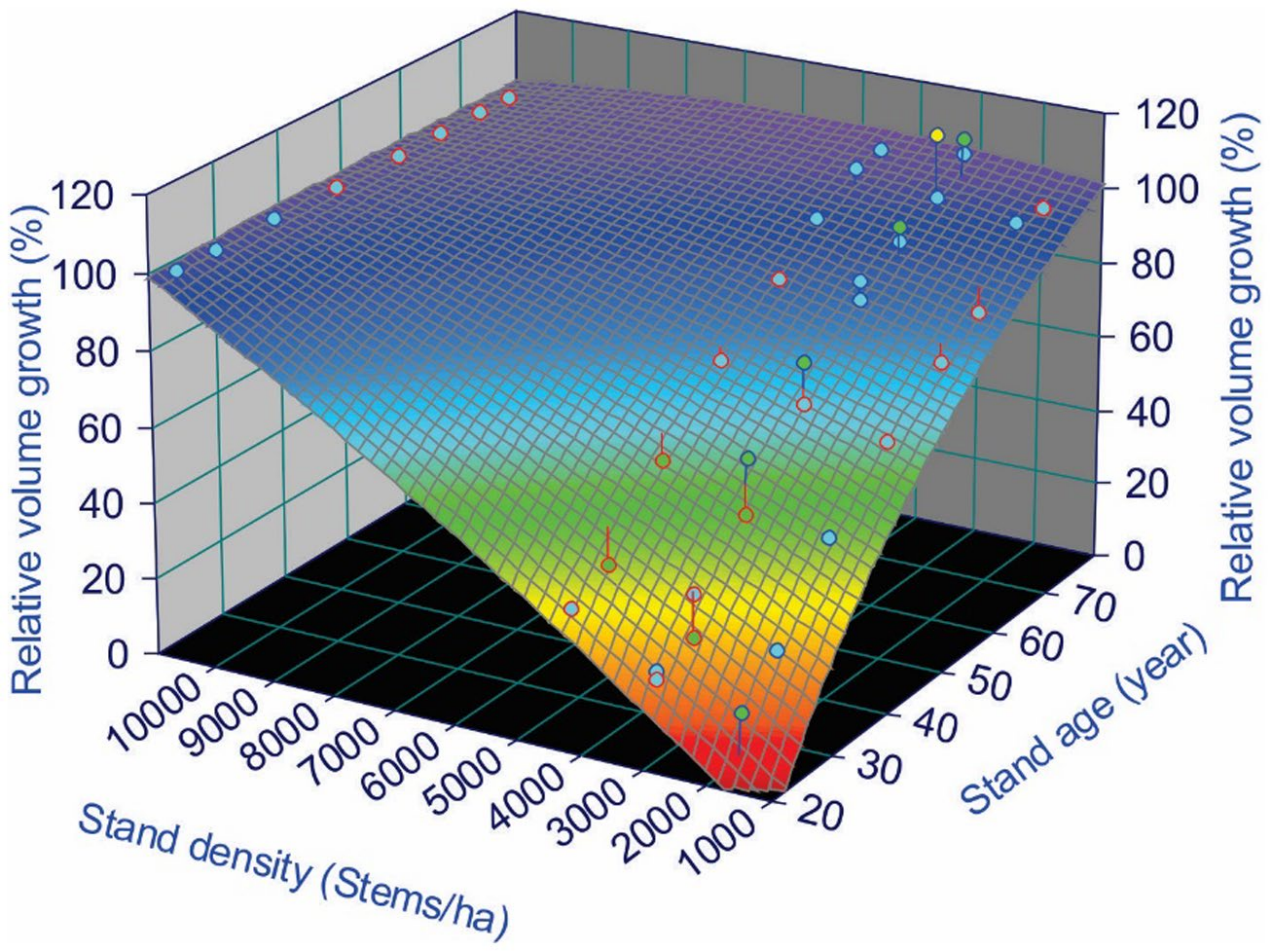
Compensatory growth (CG) of lodgepole pine (*Pinus contorta* Douglas) in Block A of the MacKay thinning trial in Alberta, Canada, expressed as relative volume growth under different stand densities over a 60-year experimental period. Maximum relative volume growth was calculated as the ratio of the volume obtained in the treated vs. control stands (see Eq. 1).

A 3D surface plot using a smoothing function for relative volume growth under different stand densities and ages shows the trend of CG that occurred over the study area. From Table 2, we observe that almost all plots from Block C reached exact or overcompensation, and that all plots from Block B were below 100% compensation. Plots from Block A were a mix of under, exact and overcompensation. Therefore, as an example of such a 3D surface plot, Figure 2 shows the data from the Block A of the MacKey thinning trial.

Figure 2 shows a clear CG pattern, in which relative volume growth was low immediately after the PCT treatments (age 20 years), but gradually approached control over time. Around 60 years after the PCT, plots with a stand density of 4,330 and 2,990 stems/ha reached CIE, but for plots with stand density of 1,680 stems/ha, the RG was still below 100% (undercompensation).

**Figure 3:**
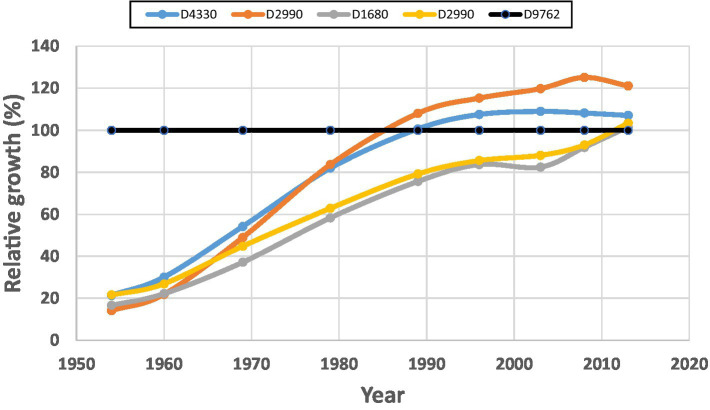
Compensatory growth patterns for different sample plots within Block C of the MacKay thinning trial in Alberta, Canada, expressed as relative volume growth under different stand densities. Maximum relative volume growth was calculated as the ratio of the volume obtained in the treated vs. control stands (see Eq. 1). Data shows that plots with a same stand density can reach compensatory-induced-equity (CIE) by different paths.

We also observed different CG patterns among individual plots, though they are physically located very close. For example, Figure 3 illustrates data from Block C, in which two plots with a same stand density (2,990 stems/ha) reached CIE from different paths; one plot reached CIE 25 years earlier than the other one. This stressed the need to pay attention to the different growth rates, or the different responses to partial mortality, among individual trees.

### Case Study 2 (Red Pine): Relative Volume Indicator Using Metadata

There is much empirical evidence to support the theoretical expectation that thinning maintains remaining trees that are vigorous and less prone to breakage at the individual tree level (e.g., Reukema and Bruce, 1977; Greene and Emmingham, 1986; Kim et al., 2016; Gauthier and Tremblay, 2019; Gupta et al., 2020; Bjelanovic et al., 2021). However, empirical evidence supporting the expectation of overcompensation are scares. There are few long-term datasets from CT trials to support it.

**Figure 4:**
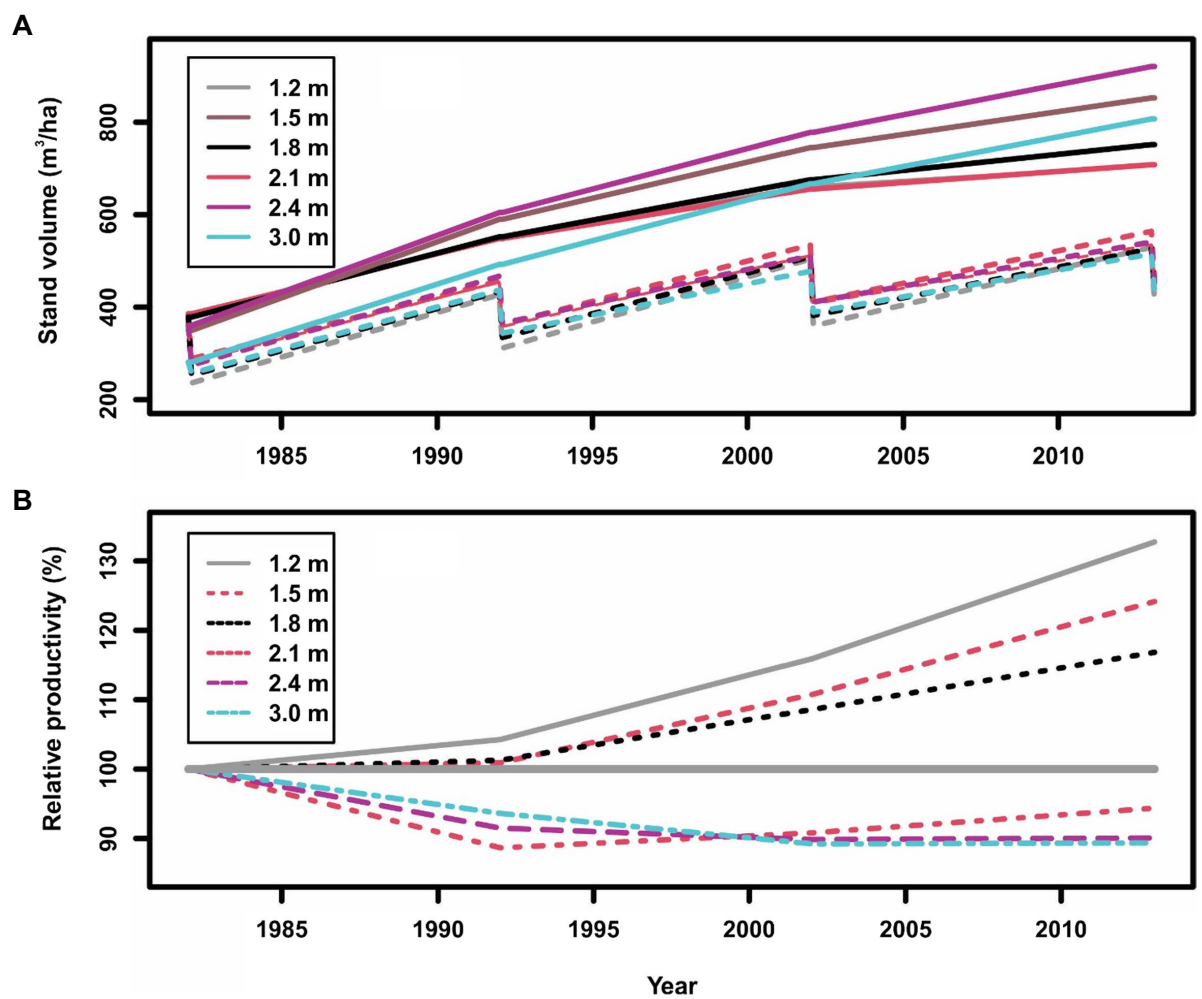
Stand gross volumes (**A**: in which solid lines indicate control plots, and dashed lines indicate thinned plots) and relative productivity (volume growth) (**B**: in which the line of 100% indicates the control plots, >100% indicates overcompensation, and <100% indicates under compensation) of planted red pine (*Pinus resinosa* Aiton) under different spacing regimes created by initial planting density and thinning in Chalk River, Ontario, Canada.

Plotting raw data of gross volume over time from the red pine experiment displays the shapes of stand growth trajectories of both thinned and unthinned plots under different spacing regimes. One might expect that all thinned plots will show a “Chainsaw” shape (see Figure 4). When displayed in cumulative stand productivity, which is the standing volume plus harvested volumes previously, this allows a comparison with stand volumes from unthinned plots. This comparison could be better represented with relative volume growth.

As reported by Thiffault et al. (2021), the stand volumes between treated and untreated plots at the beginning of the CT trial were different; thus, we scaled stand volumes to the same level at the beginning of each spacing treatment for an easy comparison. Two points can be observed from Figure 4:

1. As expected, the normal (solid lines) and “Chainsaw” (dashed lines) shapes of standing volumes from unthinned and thinned plots, respectively, are evident in Figure 4A. The Chainsaw shape illustrates that standing volume declined right after each thinning operation, and then gradually increased until the next thinning operation for a similar cyclic fluctuation. However, it is still difficult to judge if the increase of standing volume reached a level of overcompensation.
2. The relative productivity (cumulative volume growth) under different spacing regimes (Figure 4B) clearly indicate overcompensation when spacing was narrower than 1.8 m, and under compensation when spacing was wider than 2.1 m. This appears consistent with what was reported by Thiffault et al. (2021), who showed that cumulative harvested and standing volume in narrow spacing treatments exceeded that from corresponding control plots. Thiffault et al. (2021) explained this as being too low stand density that resulted in insufficient utilization of available resources. Nevertheless, relative productivity further displays different CG patterns under different spacing regimes.

The second point above may also provide a possible explanation for the so-called “initial shock,” a negative impact of a thinning operation, of the coastal Douglas-fir after a thinning operation reported in Harrington and Reukema (1983), in which stand volumes in thinned stands were observed as reduced compared with unthinned stands, 25 years later. The initial shock could be caused by sudden exposure to high wind and light, leading to moisture stress and, greater allocation of growth to roots. There is also likely mechanical damage to roots of residual trees during harvest; however, it could also probably caused by too wide spacings (3.4–8.1 m, corresponding to 875–125 stems/ha) for poor site conditions (height at 100 years = 24 m).

In addition in displaying diverse CG patterns in the red pine plantations, this case study also provides an empirical evidence of how proper CT regimes can enhance stand productivity. It appears that an increase in stand density could result in more enhanced stand productivity; however, a question remains on whether maximal stand productivity could be reached when stand density increases to a maximum. This CT trial could not provide a full answer to this question, as the maximal stand density was only covered until 6,944 stems/ha.

### Case Study 3 (Douglas-fir): Volume and Value-Based Indicators Using Tree Data

The Shawnigan Lake trial has tree-based records for all treated and control plots, with multiple measurements over 40-years. The dataset has spawned a large number of publications over the past 4 decades from a variety of perspectives and specific analyses. Earlier results have shown that increased growth has been happening in some treatments. For example, Brix (1993) showed that 15 years after the initial treatment, stand volume in some treatments exceeded those from control plots. Using the volume estimates from these reports, Li et al. (2018) used relative volume growth to demonstrate that the volumes in more than half of the treated plots had reached exact compensation by 24 years after the initial treatments (measured in 1994). Around 40 years after initial treatments, most of treatment plots have reached overcompensation, except plots without fertilization. This illustrated how the CG process happened over the 40-year’s horizon, from under compensation to CIE and to overcompensation, and how diverse CG patterns resulted from the different treatments.

Furthermore, a two-way ANOVA analysis on the 40 year data indicated that fertilization contributed significantly to stand volume differences (*p* = 0.015), whereas thinning (*p* = 0.616) and the fertilization × thinning did not (*p* = 0.587; Li et al., 2018). This result suggested that fertilization was the major factor behind the diverse CG patterns.

In the current study, we aimed at making full use of tree level records from the 2012 measurements to evaluate different value-based estimates as additional indicators of CG, and compare them with gross volume results for examining their relative sensitivity in detecting overcompensation.

#### Commodity Value-Based CG Indicator

Commodity values are the most direct and explicit way of representing wood value potential for a given forest inventory. For a volume-based commodity such as pulp, one could expect that the value recovery is proportional to volume recovery. However, for a dimensional commodity such as lumber, the situation could differ.

**Figure 5:**
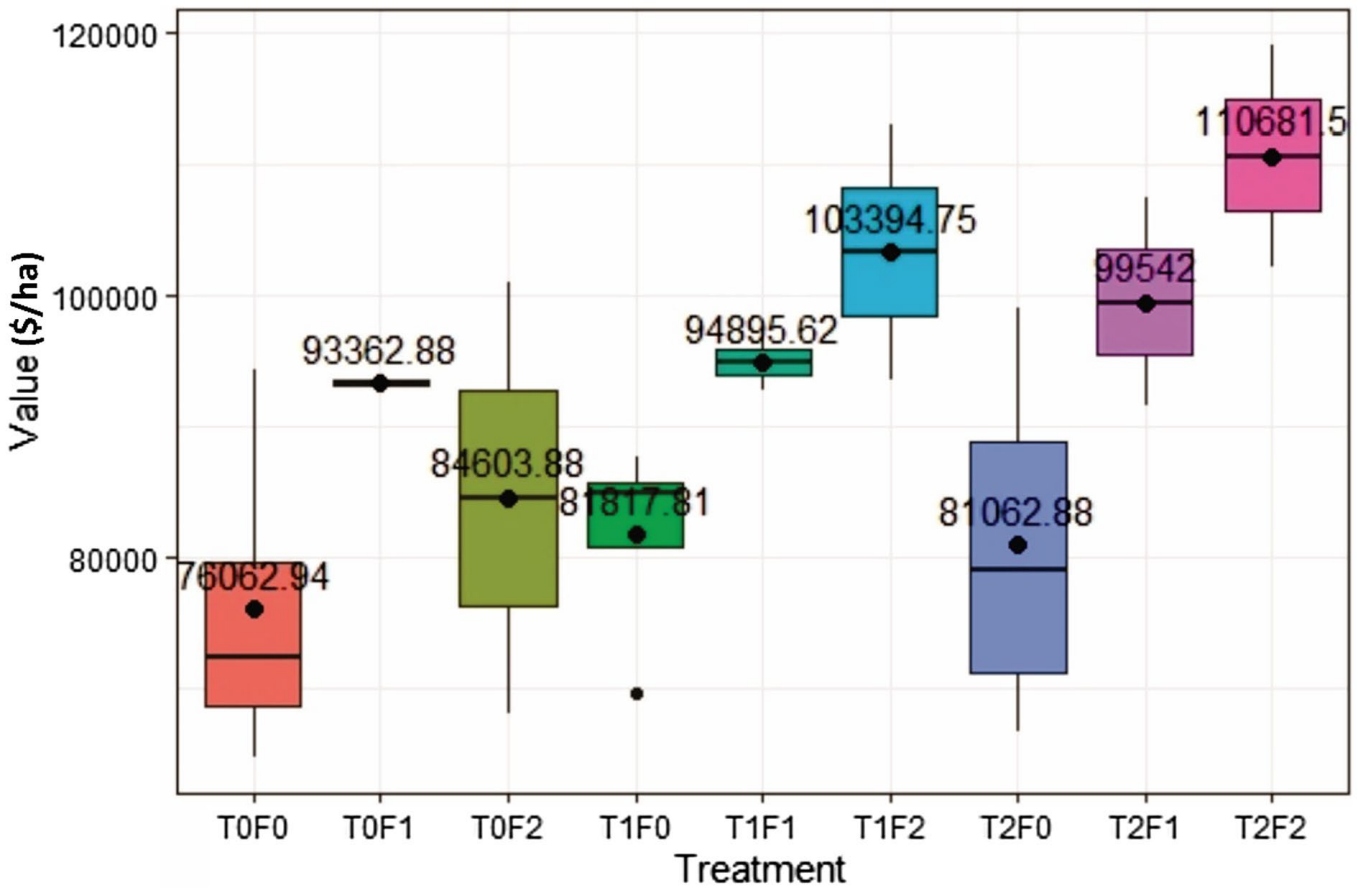
Simulated lumber value recovery for Douglas-fir (*Pseudotsuga menziesii* Mirbel) trees as a function of different combinations of pre-commercial thinning (PCT) and fertilization, 40 years after initial treatments at the Shawnigan Lake trial in British Columbia, Canada. T0, T1, and T2 correspond to 0, 1/3, and 2/3 basal area removal, respectively. F0, F1, and F2 correspond to 0, 224, and 448 kg N/ha fertilization with urea, respectively.

Lumber value recovery from all treated sites exceeded that from the untreated site (T0F0; no thinning and no fertilization), ranging from $4,999 to $34,618/ha on average 40 years after treatments (Figure 5).

The two-way ANOVA (Table 3) showed that fertilization increased stand lumber value recovery significantly. Pre-commercial thinning and the fertilization × thinning interactions did not influenced stand lumber value. A commodity value-based assessment displayed the same trend to that from a volume-based assessment (Li et al., 2018). In other words, fertilization could have contributed more in lumber value recovery increase than that of PCT, which is also consistent with the results from another fertilization study conducted in the same region (Cosmin Filipescu, Pers. Comm. Canadian Forest Services, Jan., 2020). Since fertilization has been treated as a surrogate of site quality, the ANOVA results also suggested that acceleration of the CG process could be expected with improved site quality.

**Table 3:** Two-way ANOVA results for lumber value recovery of Douglas-fir (*Pseudotsuga menziesii* Mirbel) trees under different treatments, 40 years after application at the Shawnigan Lake trial in British Columbia, Canada.

Effects (fixed)	DF	Sum Sq	Mean Sq	*F* value	Pr(>F)
Thinning	2	484,605,043	242,302,522	1.561	0.242
Fertilization	2	2,004,875,596	1,002,437,798	6.458	0.009^**^
Thinning × Fertilization	4	359,216,108	89,804,027	0.579	0.683
Residuals	15	2,328,232,519	155,215,501		

*DF = degrees of freedom*.

** *Significant at *α* = 0.01*.

A commodity value-based CG indicator in monetary term is easy to understand and effective in decision support; however, this indicator could be influenced by market fluctuations in commodity price. For example, lumber value per thousand board feet (Mfb) can change quickly and with great amplitude. The lumber value used in the current Optitek simulation was set as $400/Mfb, although in the real market it can be as high as $1,600/Mfd when market demand is high, and as low as $250/Mfd when market conditions are unfavorable. As a result, one could expect that relative value could improve representation.

#### Relative Value-Based CG Indicator

**Figure 6:**
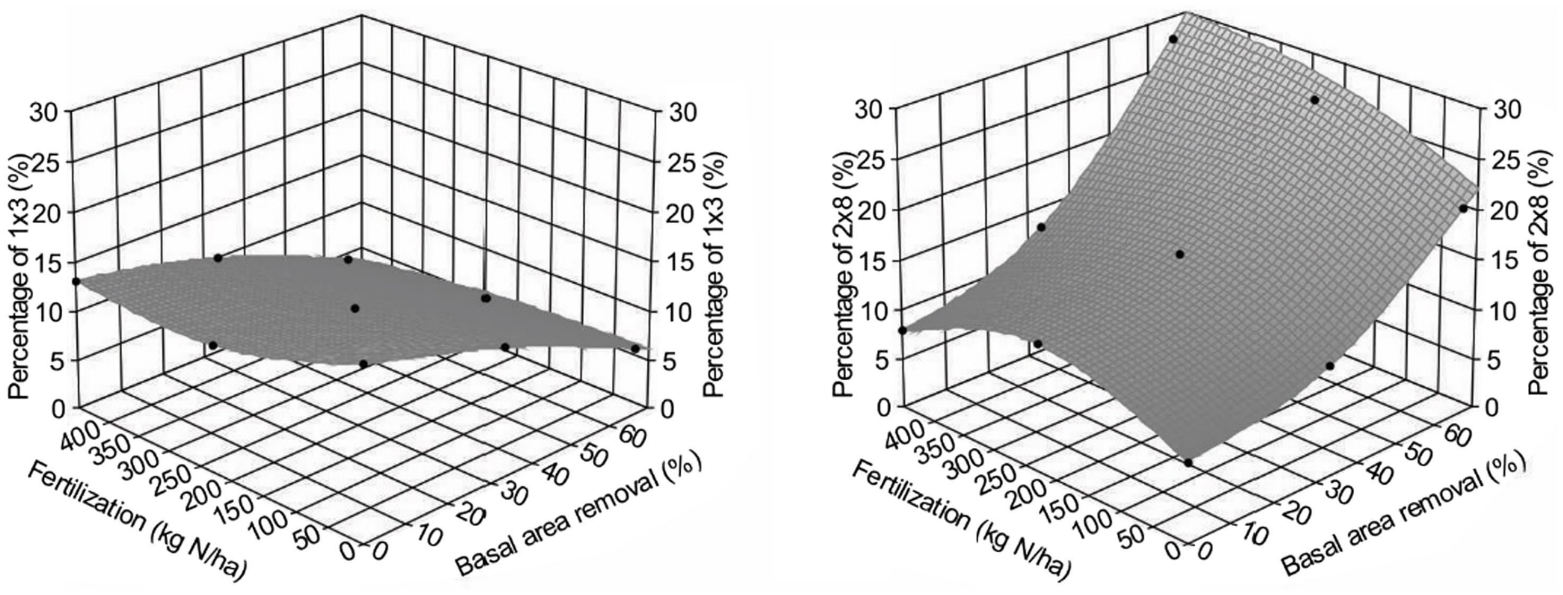
Effect of different treatments combinations on the percentage of small (left, represented by 1 inches × 3 inches lumber) and large (right, represented by 2 inches × 8 inches lumber) dimension lumbers of Douglas-fir (*Pseudotsuga menziesii* Mirbel) trees, 40 years after application at the Shawnigan Lake trial in British Columbia, Canada.

**Figure 7:**
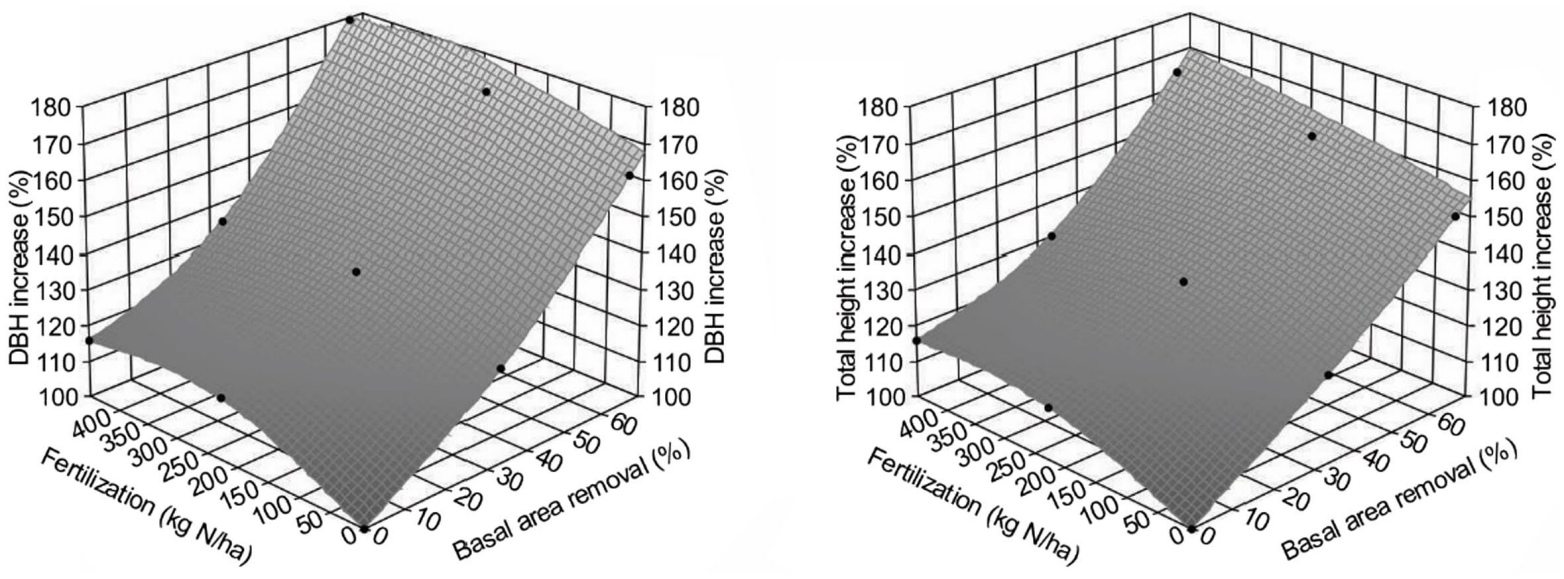
Effect of different treatments combinations on average increase of diameter at breast height (DBH; 1.3 m; left) and total height (right) for Douglas-fir (*Pseudotsuga menziesii* Mirbel) trees, 40 years after application at the Shawnigan Lake trial in British Columbia, Canada.

Figure 5 shows that PCT increased lumber value recovery, and that fertilization amplified the impact significantly (Table 3). Different from gross volume-based assessment in Li et al. (2018), lumber value recovery from unfertilized sites also exceeded those from control sites ($5,000/ha for T1F0 and $4,999/ha for T2F0). This surprising result could be explained by the changing percentage of small and large dimension lumber (Figure 6), in which increased percentage of large dimension lumber (usually worth more than small dimension lumber) under increased PCT intensity resulted in the total value recovery increase unproportioned to volume. This non-intuitive result could also be further explained by the changes in average tree size under PCT and fertilization (Figure 7); trees with larger sizes favor the production of large dimension lumbers.

**Figure 8:**
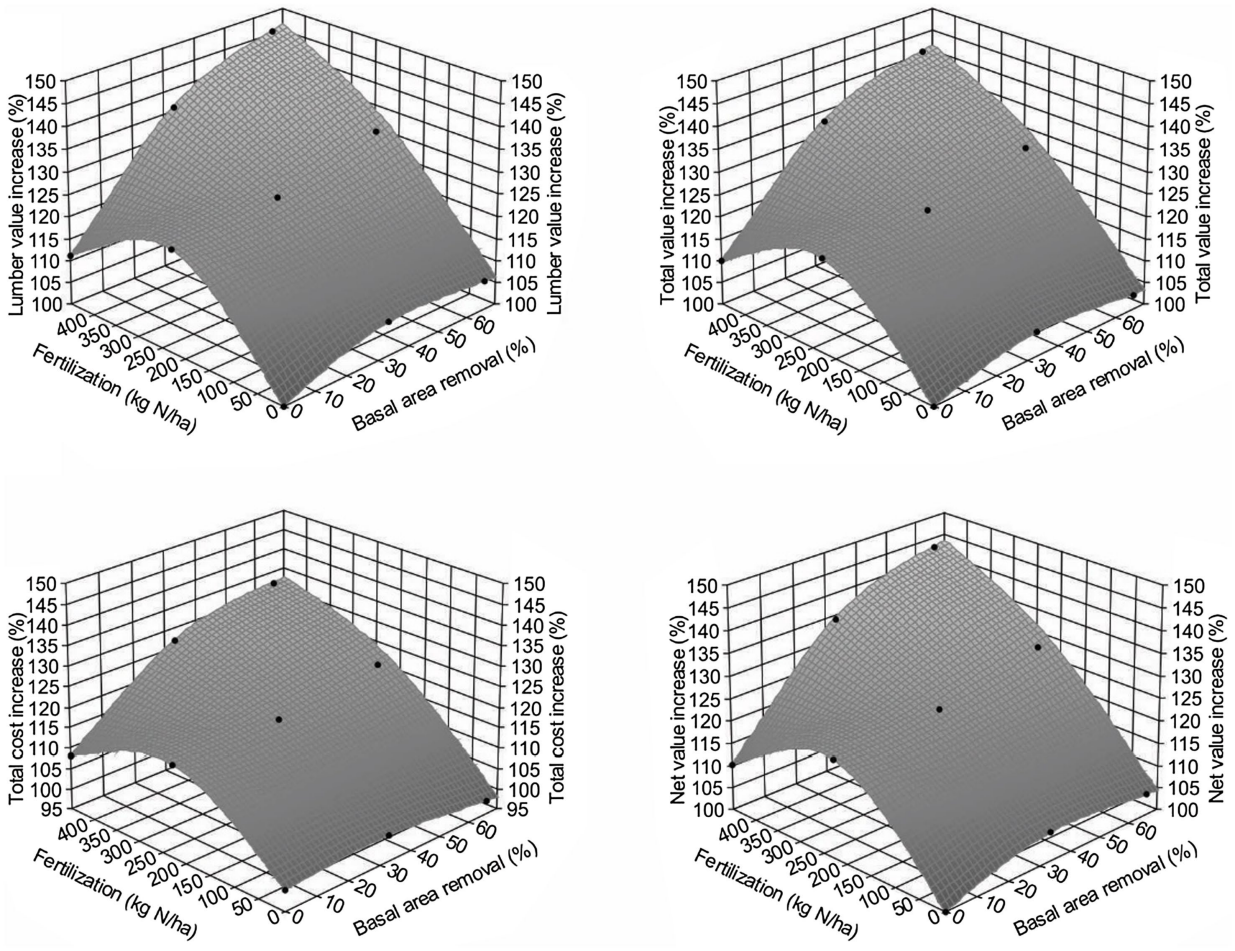
Increase of lumber value recovery (upper left), total value recovery (upper right), total operational cost (lower left), and net value recovery (lower right) for Douglas-fir (*Pseudotsuga menziesii* Mirbel) trees, 40 years after different treatment combinations relative to the untreated sites at the Shawnigan Lake trial in British Columbia, Canada.

Given the above, we estimated the trend of how lumber value recovery would change across different treatments by using the WFVSM model. The results (Figure 8) indicated that the lumber value recovery will likely increase with increasing PCT intensity and fertilization. Although relative total sawmill value recovery may not be as high as the lumber value recovery, the trend should be similar, as total production costs will also increase with the increasing PCT intensity and fertilization. As a result, the net value recovery from the sawmill operation will have a very similar pattern compared with the lumber value recovery.

The indicator of relative value recovery enables comparison with relative volume growth for identifying whether lumber value recovery based assessment would be more sensitive in detecting overcompensation than that of volume-based assessment.

#### Comparison Between Relative Volume and Value-Based Indicators

**Figure 9:**
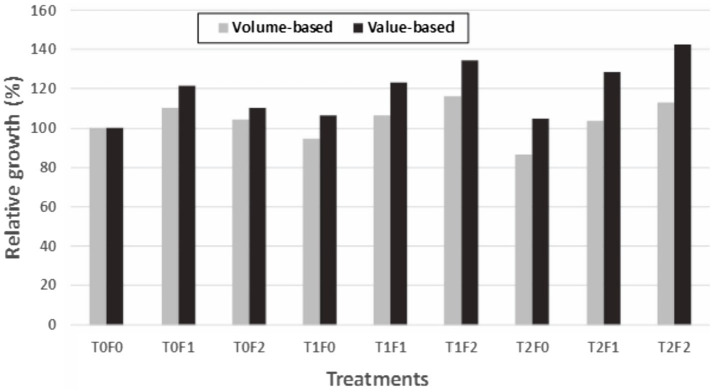
Commodity value-based vs. volume-based growth estimates for Douglas-fir (*Pseudotsuga menziesii* Mirbel) trees relative to untreated sites (as 100%) for different treatment combinations, 40 years after application at the Shawnigan Lake trial in British Columbia, Canada.T0, T1, and T2 correspond to 0, 1/3, and 2/3 basal area removal, respectively. F0, F1, and F2 correspond to 0, 224, and 448 kg N/ha fertilization with urea, respectively.

When we compared volume-based assessment as summarized in Li et al. (2018), all value-based relative growth indicators exceeded 100% (i.e., overcompensation) and are higher than those from volume-based assessment (Figure 9). This suggests that value-based indicator might be more sensitive than volume-based assessment in detecting overcompensation.

Overall, this case study shows that PCT triggered CG processes and eventually overcompensation, and that relative growth indicators allowed displaying diverse CG pattern depending largely on the level of fertilization or site quality. The intensity of PCT was employed as an independent variable for predicting the time required to reach CIE.

### Lessons From Three Canadian Case Studies

Detection of CG patterns and status is a pre-condition for designing and taking full advantage of CG phenomenon in silviculture and forest management. For example, the capacity for predicting long-term stand growth trajectories under various partial mortality scenarios could provide decision support for designing the best silviculture prescription. Forecasting levels of enhanced long-term productivity may provide a solid foundation for increasing regional annual allowable cut, thus supporting consistent wood supply as a key component of sustainable forest management (e.g., Pinno et al., 2021).

Compensatory growth appears to be a common phenomenon across taxa (Li et al., 2021). The case studies we present here suggest that CG also commonly exists in a range of tree species. The possible reasons for its not having been reported widely in the literature can be attributed to:

- Long lifespan of trees: This makes the detection of CG difficult due to the scarcity of appropriate data. This problem is not present for short lifespan species such as annual plants, animals in farm, fishery, and aquaculture, for which experimental approaches to detect CG are relatively easy to implement.
- Lack of proper methods or indicators in CG detection: researchers usually report the absolute values in stand volume; however, the comparison of results can be misleading if volumes are calculated using different methods (Li et al., 2015). RG could avoid this difficulty but is rarely used.
- Research focus and terminology: overcompensation is highly desirable in the forest sector; however, the topic is seldom an explicit research focus, primarily due to the paucity of long-term observations. The red pine dataset presented in our second case study (the red pine experiment) illustrates that CT is probably a worthy treatment to take advantage of the volume that would be loss to natural mortality otherwise (Thiffault et al., 2021). While CG was indeed documented in their study, it was not termed so.

The three case studies described here consist of three data structures (stand level density management, paired CT results, and individual level of tree records), showing that detection of CG patterns might not require high-resolution datasets, though tree records could provide more options of CG indicators. Our results show that revisiting historical datasets from long-term legacy silviculture trials, even those designed for other purposes, and using a universal indicator of RG can enable detecting CG patterns (Figure 2) and different status (Table 2), without having to wait for newly designed experiments to be completed. However, when tree-level data are available, both volume and value-based indicators could be used, and lumber value related indicators are more sensitive to overcompensation (Figure 9).

Insufficient replicates might influence the ability to generalize conclusions. For example, Figure 3 shows different paths to CIE for two plots with a same stand density located close to each other. Though this can be attributed to different individual responses to the same disturbance, it also suggests that enough replications is necessary for avoiding inaccurate general conclusions.

Also, a lack of coverage of a large gradient of site quality might pose challenges, because different site conditions may alter the number of years necessary to reach CIE. For example, the datasets used in the first (MacKay experiment) and third (Shawnigan Lake experiment) case studies reported here originate from poor sites, while the dataset from the second (Petawawa Research Forest experiment) case study originates from a good site, exhibiting a top performance for the species. They are theoretically unsuitable for a direct comparison, thus creating difficulties for developing a generalized equation for predicting the number of years to reach CIE. Nevertheless, it suggests that the probability of forest productivity to be enhanced by PCT or CT could be higher on good sites than on poor sites.

Despite these limitations, historical legacy projects are interesting data sources for detecting CG in forests stands. Furthermore, our analyses also suggest that a modeling approach might be a more favorable way than a strictly experimental approach to improve our understanding of CG and our capacity to predict CG in forest ecosystems.

## Conclusion and Recommendations

Our three case studies using historical legacy silviculture datasets suggest that CG might be common in tree biology and forestry. Relative growth could serve as a universal indicator for detecting CG in forest ecosystems; when tree data are available, both volume- and value-based indicators can be used for this purpose, and lumber-value related indicators could be more sensitive to overcompensation than other indicators. However, assessment could be complicated by other factors that influence forest productivity, such as age structure of stands and species composition.

Due to the long lifespan of trees, experimental approaches might not be as efficient in detecting CG as for other plants and animals with shorter lifespan. Therefore, a modeling approach could be employed to accelerate our understanding and prediction of CG in tree biology and forestry for decision support in forestry practice.

## Data Availability Statement

The data analyzed in this study is subject to the following licenses/restrictions: Permission of the datasets used in this study obtained from data providers. Requests to access these datasets should be directed to Jim Stewart; NT; and Cosmin Filipescu.

## Author Contributions

CL, HB, and SH: research design, data analysis, and draft writing. CL, HB, SH, BR, RL, and NT: review and editing. All authors contributed to the article and approved the submitted version.

## Funding

This work was financially supported by Natural Resources Canada-Canadian Forest Service’s Developing Sustainable Fibre Solutions Research Program.

## Conflict of Interest

The authors declare that the research was conducted in the absence of any commercial or financial relationships that could be construed as a potential conflict of interest.

## Publisher’s Note

All claims expressed in this article are solely those of the authors and do not necessarily represent those of their affiliated organizations, or those of the publisher, the editors and the reviewers. Any product that may be evaluated in this article, or claim that may be made by its manufacturer, is not guaranteed or endorsed by the publisher.
